# Sensory and cognitive awareness impairment patterns in children with autism spectrum disorder: a factorial analysis of the underlying constructs

**DOI:** 10.1186/s13034-025-00967-5

**Published:** 2025-10-15

**Authors:** Worku Abie Liyew, Ayalew Moges, Fikirte Girma, Workeabeba Abebe, Mekbeb Afework

**Affiliations:** 1 Department of Anatomy, School of Medicine, College of Health Science, Addis Ababa University, Addis Ababa, Ethiopia; 2 Department of Pediatrics and Child Health, School of Medicine, College of Health Sciences, Addis Ababa University, Addis Ababa, Ethiopia; 3 Department of Psychiatry, School of Medicine, College of Health Sciences, Addis Ababa University, Addis Ababa, Ethiopia

**Keywords:** Autism spectrum disorder, Factor analysis, Impairments, Pattern, Sensory, Cognitive awareness

## Abstract

**Background:**

Individuals with autism spectrum disorder (ASD) have a wide range of challenges related to sensory and cognitive awareness. In Ethiopia, the increasing prevalence of ASD underscores the need for a comprehensive understanding of the associated challenges and impairments, an area that has not been studied so far.

**Objective:**

The objective of this study was to investigate the underlying patterns of sensory and cognitive awareness impairments in children diagnosed with ASD at autism centers in Addis Ababa, Ethiopia.

**Methods:**

An institution-based cross-sectional study was conducted at the Nehemia Autism Center and the Nia Foundation in Addis Ababa, Ethiopia. The study included children aged 4 to 16 years who had a confirmed diagnosis of ASD. A total of 145 study participants involved in this study. Study subjects were identified in collaboration with staff and caregivers. Caregivers of the study subjects were approached by trained data collectors, and written informed consent was obtained. The sensory/cognitive awareness subscale of the Autism Treatment Evaluation Checklist (ATEC) was administered to caregivers. This questionnaire tool has been validated for the autism population in Ethiopia. A face‒to-face interview was conducted. Data analysis was conducted IBM SPSS Version 22 Statistical Software. Principal component analysis with varimax rotation was employed to examine the patterns of sensory and cognitive awareness impairments. The numbers of principal components and factors to be retained were determined by examining the Eigenvalues and scree plot. Eigenvalues greater than 1 were used. The variable composition of each factor was examined by analyzing the factor loadings in the rotated component matrix. High variable loadings above 0.3 were considered for each factor.

**Results:**

This study revealed five patterns of sensory and cognitive awareness impairments in children diagnosed with ASD. Pattern 1, limitation in social engagement and exploration (α = 0.822); Pattern 2 challenges in emotional awareness and cognitive responsiveness (α = 0.743); Pattern 3 challenges in story comprehension and creativity (α = 0.62); Pattern 4 difficulties in social reciprocity and reward (α = 0.34); and Pattern 5 trouble with focus and attention (α = 0.12). All of these patterns accounted for 60% of the total variance.

**Conclusion:**

In this study, five patterns of sensory and cognitive awareness impairments were identified. Clinicians and therapists may need to consider these patterns for more personalized and effective support of children with ASD.

## Introduction

Autism spectrum disorder (ASD) is a complex and heterogeneous neurodevelopmental disorder characterized by basic behavioral symptoms, such as difficulties in social interactions, impaired communication, and the presence of repetitive behavior or restricted interests [1–6]. In addition, people with ASD experience a diverse range of challenges related to sensory and cognitive awareness. They might find it difficult to interpret their own thoughts and the perspectives others, as well as to react to sensory information [7]. These can significantly affect their daily functioning, social interactions, and quality of life [8–11]. Cognitive awareness impairments, including difficulties with attention, memory, and executive functioning, further affect the developmental pathway of these children.

Before the 1990s, the rate of ASD was relatively stable, estimated at 4 to 5 per 10,000 individuals [12]. Studies conducted in the 1990s on preschool children in Japan, England, and Sweden reported increasing rates of autism between 21 and 31 per 10,000. The prevalence of ASD has increased in recent years and is becoming an issue of public concern. In 2006, the incidence of autism increased to 1 in 110 children, and in 2008, it again increased to 1 in 88 children. In 2012, the autism and developmental disabilities monitoring (ADDM) network revised its autism estimates to 1 in 68 children [13]. In 2016, the National Center for Health Statistics (NCHS) released its latest rate, reporting a high record of autism that affects up to 1 in 36 children [14]. Improved screening procedures, better diagnostic standards, and increased awareness are some of the reasons for this increase [15–17].

Autism is not well understood in Africa, including Ethiopia, and most related studies have been conducted in developed countries [18]. A conference conducted on ASD in Africa from 14 countries reported a lack of awareness of identification, diagnosis, management and delivery support for individuals with ASD across the country and highlighted the need to consider culture, linguistic, and socioeconomic factors [19].

In Ethiopia, an estimate made in 2002 by the Nia Foundation estimated that possibly 500,000 children had autism. A recent estimation by the foundation in 2015 indicated that approximately 530,000 children have autism and related developmental disorders. The increasing prevalence of autism in Ethiopia indicates the importance of advancing our understanding of various aspects of ASD. In Ethiopia, most existing research has focused on parental burdens, experiences, coping strategies, and caregiving practices [20–26]. There is insufficient evidence documenting the specific sensory and cognitive awareness impairment patterns affecting children diagnosed with autism. Moreover, the available data originate primarily from high-income countries and middle-income countries and may not be directly applicable to the Ethiopian context [18, 27]. This highlights the need for localized research that considers the cultural, environmental, and socioeconomic factors that influence autism in Ethiopia. Therefore, this study aimed to investigate the underlying patterns of sensory and cognitive awareness impairments in children diagnosed with ASD at autism centers in Addis Ababa, Ethiopia.

## Methods and materials

### Setting, design and study period

An institution-based cross-sectional study was carried out among children diagnosed with ASD at the Nehemia Autism Center and the Nia Foundation, Addis Ababa, Ethiopia. Data collection was conducted from June to October 2024.

### Study participants

All children diagnosed with ASD and between the ages of 4 and16 years were the source population. Children with a confirmed clinical diagnosis of ASD who met the inclusion criteria composed the study population. In Ethiopia, the mental health clinic recruited children with ASD in accordance with the Diagnostic and Statistical Manual of Mental Disorders, Fifth Edition (DSM-5). No standardized diagnostic tests are available in Ethiopia; therefore, clinicians relied on their clinical judgment based on clinical interviews with the caregiver and observation and interaction with the child for the neurodevelopmental assessments. During data collection, a diagnosis certificate was verified for each study participant. No independent diagnostic reassessment was performed. Children without a clinical diagnosis of ASD were excluded. Caregivers or parents who declined to give their consent were excluded. Furthermore, study participants with other neurological comorbidities, such as seizures or those on medication, were also excluded.

### Sample size determination and sampling technique

The sample size was determined using the subject-to-variable ratio for factor analysis. The calculation adhered to the subject-to-variable ratio for factor analysis 5:1 (Rule of 5) with a minimum sample size of 90 [28]. A total of 177 children diagnosed with ASD were found at the two autism centers. Based on the established inclusion criteria of this study, a maximum of 145 children were included. Consecutive sampling was used on the basis of the presence of caregivers for the autistic children in the classroom.

### Data collection

#### Identification, recruitment, and ethical enrollment of participants

Initially, the principal investigator approached the staff and caregivers of children with ASD at the Nehemia Autism Center and the Nia Foundation. They were provided with detailed information about the purpose, procedures, potential risks and benefits of the study. The study subjects were subsequently identified and recruited in collaboration with the staff and caregivers. Caregivers of study subjects were approached by trained data collectors. Written informed consent was obtained from the caregivers of the participating children. The participants were assured that their participation was voluntary and that they could withdraw at any time during data collection. To ensure compliance with ethical standards, the study protocol was reviewed and approved by the institutional review board (IRB) of the College of Health Sciences, Addis Ababa University, and reference number 097/23/Anat was provided.

#### Procedure

The Amharic version of the Sensory/cognitive awareness subscale of the Autism Treatment Evaluation Checklist (ATEC) [29] was administered. The ATEC has been validated for use in the autism population in Ethiopia [30] and was therefore used in this study. The questionnaire was administered through a face‒to-face interview lasting approximately 10–15 min. The interviews were conducted in private rooms at the centers to ensure confidentiality and minimize distractions. Data collectors read the questions to the caregivers and recorded their responses. The principal investigator facilitated the process, ensuring that caregivers understood the sensory/cognitive awareness subscale of ATEC and could provide accurate responses.

*ATEC* ATEC was developed by Bernard Rimland and Stephen M. Edelson of the Autism Research Institute. It consists of 77 items that evaluate individual behaviors of ASD across four subscales: (I) speech/language/communication (14 items, score range from 0 to 28); (II) sociability (20 items, score range from 0 to 40); (III) sensory/cognitive awareness (18 items, score range 0–36); and (IV) health/physical (25 items, score range 0–75). ATEC can be completed by parents, teachers, or others who see the individual’s behavior with ASD. This questionnaire tool is used to measure the degree of clinical improvement and severity of symptoms associated with ASD [31–34], as well as to help parents identify whether their children could benefit from specific treatments or interventions. In the present study, the sensory and cognitive awareness subscale (18 items) was examined to identify the different patterns of impairments and group them into distinct categories. Each item was scored on a scale of 0–2 points severity, with the following classifications: 0 for “not descriptive,” 1 for “somewhat descriptive,” and 2 for “very descriptive.” For simplification, the scoring was standardized to 0 (not a problem), 1 (somewhat a problem), and 2 (a problem).

##### Operational definition

*Sensory and cognitive awareness* Sensory and cognitive awareness refer to the observable behavioral characteristics assessed by the sensory and cognitive awareness subscale of the ATEC measurement.

*Sensory and cognitive awareness impairment* This refers to challenges or deficits in behaviors evaluated by the sensory/cognitive awareness subscale of the ATEC.

*Sensory and cognitive awareness impairment patterns* These are distinct groups or clusters of difficulties identified in behaviors assessed by the sensory/cognitive awareness subscale of the ATEC, which is determined through factor analysis of caregiver responses.

###  Data analysis

Data analysis was conducted using IBM SPSS Version 22 statistical software. To explore the underlying patterns of sensory and cognitive awareness impairments, principal component analysis (PCA) and varimax orthogonal rotation methods were used. The Kaiser‒Meyer‒Olkin (KMO) measure of sampling adequacy and Bartlett’s test of sphericity were used to test the suitability of the data for factorial analysis [35], and a KMO value greater than 0.6 was considered adequate for analysis [36]. The numbers of principal components and factors to be retained were determined by examination of the Eigenvalues and scree plot, with Eigenvalues greater than 1 and the “elbow” where the eigenvalues start to level off were used [35]. The variable composition of each factor was examined using factor loading analysis in the rotational component matrix. High variable loads above 0.3 were considered for each factor [37]. For items displaying multiple cross-loadings, the item with the highest load was selected for inclusion in the final model [38]. The variables in each factor were examined and labeled according to their common meanings, with simple terms used to describe the main features of each factor [35].

## Results

### Sociodemographic characteristics

A total of 145 children diagnosed with ASD were enrolled in this study. The mean age was 9.2 years, and the majority of the study participants were males (83.4%) (Table 1).

**Table 1:** Sociodemographic characteristics of the study participants and their parents (*n* = 145)

Sociodemographic variables		Frequency	Percent
Gender of children	Male	121	83.4
	Female	24	16.6
Mean ages of children = 9.19 Median ages of children = 8			
Educational status (for parents)	Can not read and write	5	3.4
	Primary school	4	2.8
	Secondary school	49	33.8
	Diploma	34	23.4
	Degree and above	53	33.6
Marital status parents	Married	106	73.1
	Single	39	26.9
Occupation of parents (for parents)	No work	55	37.9
	Private sector	56	38.6
	Government employee	28	19.3
	Merchant	5	3.4
	Farmer	0	0
	Daily laborer	1	0.7

### Statistical descriptions of the items

The means for each item related to the sensory and cognitive awareness of the study participants ranged from 0.35 (SD = 0.707) to 1.67 (SD = 0.990) (Table 2).

**Table 2:** Percent, means and standard deviations of each item assessed for sensory and cognitive awareness (*n* = 145)

Items	Frequency			Mean	Std. Deviation
	0(%)	1(%)	2(%)		
1. Respond to own name	80	4.8	15.2	0.35	0.732
2. Respond to praise	69.7	6.9	23.4	0.54	0.850
3. Look at people and animals	77.2	9	13.8	0.37	0.715
4. Look at pictures (and TV.)	66.2	21.4	12.4	0.46	0.707
5. Does drawing, coloring, art	28.3	9.7	62.1	1.34	0.891
6. Play with toys appropriately	51	6.9	42.1	0.91	0.964
7. Appropriate facial expression	51	11.7	37.2	0.86	0.933
8. Understand stories on T.V.	40	10.3	49.7	1.10	0.945
9. Understand explanations	42.8	5.5	51.7	1.09	0.971
10. Aware of the environment	44.1	5.5	50.3	1.06	0.973
11. Aware of danger	44.8	2.1	53.1	1.08	0.990
12. Show imagination	15.2	2.8	82.1	1.67	0.727
13. Initiate activities	37.9	4.8	57.2	1.19	0.960
14. Dress self	37.2	11.7	51	1.14	0.933
15. Curious, interested	46.2	2.8	51	1.05	0.988
16. Venturesome-explore	50.3	4.8	44.8	0.94	0.977
17. “Tuned in”/not spacey	46.2	7.6	46.2	1.00	0.965
18. Looks where others are looking	46.2	5.5	48.3	1.02	0.975

Scores range from 0: not a problem; 1: sometimes a problem; 2: it is a problem

### Sample adequacy tests and correlation analysis

The KMO value was 0.802, which indicates that the data were suitable for factorial analysis. This finding was also supported by the result of the Bartlett chi-square test, 860.660, which was statistically significant (*p* < 0.001) (Table 3). Therefore, the null hypothesis that items in the sensory and cognitive domains do not correlate was rejected.

**Table 3:** KMO and bartlett’s tests

Kaiser‒Meyer‒Olkin Measure of Sampling Adequacy		0.802
Bartlett’s Test of Sphericity	Approx. Chi-Square	860.660
	Df.	153
	Sig.	*p* < 0.001

### Principal component analysis

**Fig. 1:**
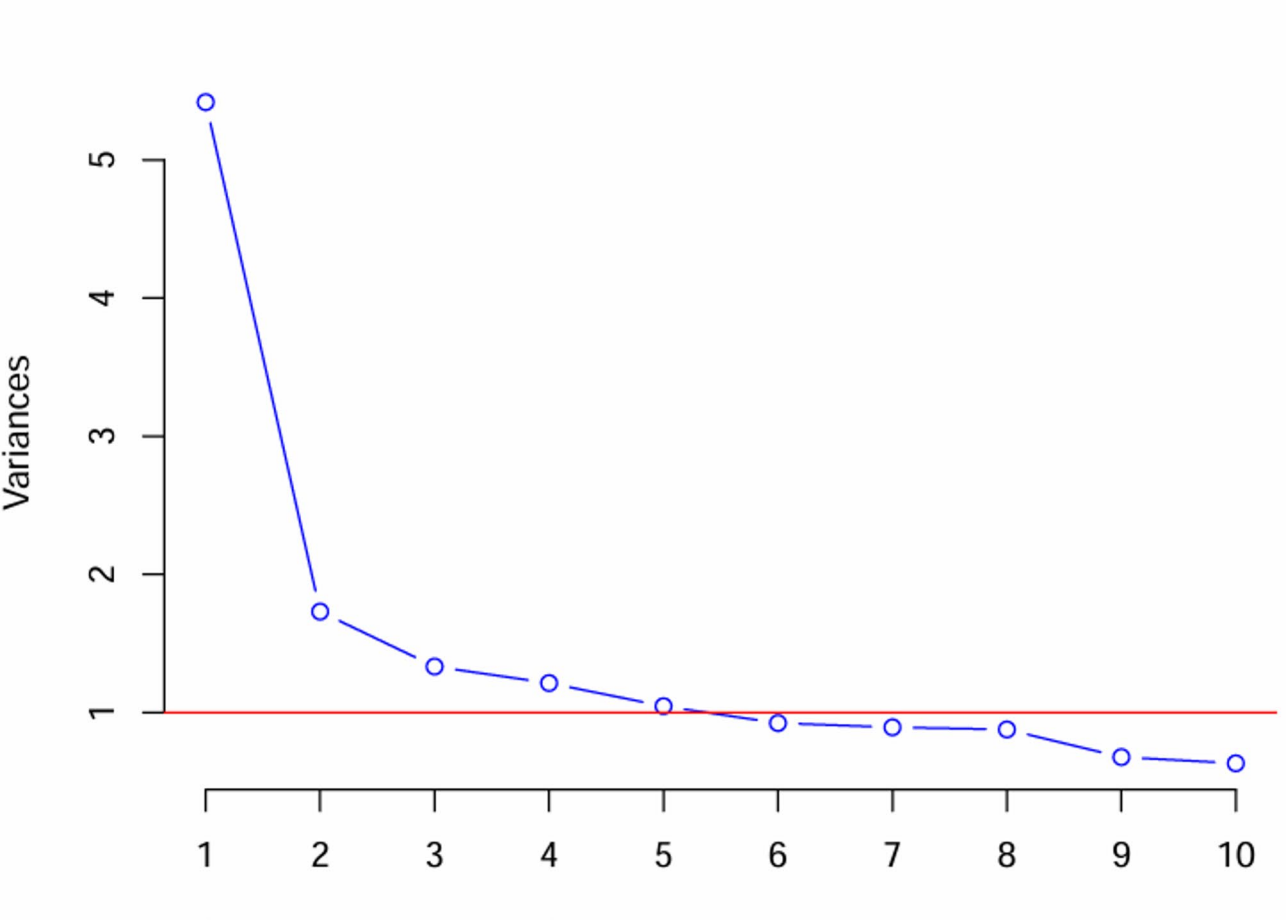
A scree plot illustrating the variances associated with the principal components. The x-axis represents the principal components (1 to 10). The y-axis shows the variances associated with each component. The blue line connects the variance values for each component. The red horizontal line represents the threshold for retaining components (typically set at 1)

An examination of the eigenvalues and a scree plot indicated that five principal components must be retained. These factors accounted for nearly 60% of the total variance. The Eigenvalues and percentages of variance for these components were 5.4 (30%), 1.7 (10%), 1.3 (7%), 1.2 (7%), 1.2 (7%), and 1 (6%), respectively (Table 4 and Fig. 1).

**Table 4:** Eigenvalues and variance explained by the PCA components

Components	Initial Eigenvalues			Rotation Sums of Squared Loads		
	Total	% of Variance	Cumulative %	Total	% of Variance	Cumulative %
1	5.418	30.099	30.099	3.415	18.971	18.971
2	1.730	9.612	39.711	2.847	15.815	34.787
3	1.333	7.403	47.115	1.609	8.938	43.725
4	1.213	6.739	53.854	1.571	8.727	52.452
5	1.046	5.810	59.664	1.298	7.213	59.664
6	0.923	5.130	64.794			
7	0.893	4.959	69.753			
8	0.877	4.873	74.626			
9	0.677	3.762	78.388			
10	0.632	3.512	81.901			
11	0.585	3.249	85.149			
12	0.539	2.995	88.144			
13	0.505	2.803	90.947			
14	0.466	2.588	93.535			
15	0.423	2.348	95.883			
16	0.355	1.974	97.857			
17	0.245	1.360	99.216			
18	0.141	0.784	100.000			

### Factor structure, naming, and loading

The rotated component matrix in Table 5 presents the patterns of sensory and cognitive awareness impairments, loading coefficients, and a common name that can describe each pattern. The analysis revealed five distinct patterns: Pattern 1, *limitation in social engagement and exploration* (α = 0.822); Pattern 2, *challenges in emotional awareness and cognitive responsiveness* (α = 0.743); Pattern 3, *challenges in story comprehension and creativity* (α = 0.62); Pattern 4, *difficulties in social reciprocity and rewards* (α = 0.34); and Pattern 5, *trouble with focus and attention* (α = 0.12). All of these patterns accounted for 60% of the total variance.

Items *16 (Venturesome-explore)*,* 15 (Curious*,* interested)*,* 4 (Look at pictures and TV)*,* 18 (Looks where others are looking)*,* 14 (Dress self)*, and *13 (Initiate activities)* were loaded more strongly to Pattern 1. In Pattern 2, five correlated items were identified: 11 (*Aware of danger*), 9 (*Understand explanation*), *10 (Aware of the environment)*,* 7 (Appropriate facial expression)*,* and 12 (Show imagination).* The third Pattern included three items: *8 (Understand stories on TV.)*,* 5 (Does drawing*,* coloring*,* art)*,* and 6 (Play with toys appropriately).* Items *2 (Responding to praise) and 1 (Responding to his/her own name)* were loaded into Pattern 4. Pattern 5 was loaded with items 3 (*Look at people and animals)* and *item 17 (Tuned in”/not spacey).* All the items had high loadings ranging from 0.49 to 0.84.

**Table 5:** Rotated component matrix from a principal component analysis (PCA) with orthogonal varimax rotation

Items	Patterns					Communalities	Cronbach’s alpha
	1	2	3	4	5		
	Limitations in social engagement and exploration	Challenges in emotional awareness and cognitive responsiveness	Challenges in story comprehension and creativity	Difficulties in social reciprocity and rewards	Trouble with focus and attention		
16. Venturesome-explore	**0.836**	0.176	0.098	0.265	− 0.150	0.832	0.822
15. Curious, interested	**0.794**	0.118	0.149	0.232	− 0.055	724	
4. Look at pictures (and T.V.)	**0.674**	− 0.022	0.091	− 0.150	0.301	0.577	
18. Looks where others are looking	**0.576**	0.355	0.131	− 0.205	− 0.195	0.555	
14. Dress self	**0.574**	0.150	0.313	0.338	0.286	0.646	
13. Initiate activities	**0.537**	0.377	0.017	0.131	0.215	0.494	
11. Aware of danger	0.046	**0.795**	0.069	− 0.078	0.195	683	0.743
9. Understand explanations	0.164	**0.732**	− 0.057	0.134	− 0.038	0.585	
10. Aware of the environment	0.146	**0.684**	0.172	0.072	− 0.031	525	
7. Appropriate facial expression	0.135	**0.590**	− 0.157	0.426	− 0.019	0.573	
12. Show imagination	0.088	**0.458**	0.405	0.232	− 0.221	485	
8. Understand stories on T.V.	0.039	− 0.087	**0.781**	− 0.121	0.128	650	0.62
5. Does drawing, coloring, art	0.262	0.091	**0.526**	0.415	− 0.063	530	
6. Play with toys appropriately	0.244	0.407	**0.487**	0.136	− 0.006	480	
2. Respond to praise	0.023	0.144	0.117	**0.702**	0.142	548	0.34
1. Respond to his/her own name	0.492	0.033	− 0.098	**0.515**	− 0.040	519	
3. Look at people and animals	0.177	0.153	0.141	0.145	**0.766**	683	0.12
17.“Tuned in”/not spacey	0.449	0.299	0.244	0.044	**− 0.545**	650	
Eigenvalue	3.415	2.847	1.609	1.571	1.298		
Explained variance %	18.971	15.815	8.938	8.727	7.213		
Cumulative %	18.971	34.787	43.725	52.452	59.664		

Top five patterns of sensory and cognitive awareness impairments, labeled 1 through 5, each representing a distinct underlying pattern. The significant variables associated with each factor are displayed in bold. Extraction Method: PCA; Rotation Method: Varimax with Kaiser normalization

## Discussion

Numerous studies have identified various brain regions associated with ASD [39–42], including the frontal and temporal cortices, caudate nucleus, cerebellum, parietal cortex, anterior cingulate cortex (ACC), basal ganglia, and amygdala. Evidence suggests that reduced functional connectivity [39, 43, 44] and atypical brain development during the first year of life [45–47] contribute to the onset and progression of ASD. Behavioral impairments in sensory processing and cognitive awareness are thought to be influenced by genetic factors and neurodevelopmental origins. These factors have implications for both clinical practice and the scientific understanding of ASD [48]. Identifying different patterns of impairment and their corresponding variable compositions is important for guiding interventions and management strategies [49]. These insights are important in customizing intervention approaches to meet the unique needs of individuals for personalized care. Scientifically, they may provide information on the fundamental causes of ASD [50], shedding light on the complex interplay of genetic, environmental, and neurobiological factors that contribute to the manifestation of different patterns.

In the present study, five distinct patterns of sensory and cognitive awareness impairments were identified. These patterns include limitations in social engagement and exploration, challenges in emotional awareness and cognitive responsiveness, challenges in story comprehension and creativity, difficulties in social reciprocity and rewards, and trouble with focus and attention. The findings of this study indicate that the observable behavioral characteristics assessed by the sensory and cognitive awareness subscale of ATEC can be represented by five underlying factors, each characterized by multiple variable loadings [35, 51]. This finding is supported by Lai et al. [52]. , who identified multiple atypical cognitive patterns in individuals with autism. These patterns include impaired social cognition and social perception, executive dysfunction, and atypical perceptual and information processing.

Each pattern of sensory and cognitive awareness impairments offers insight into the underlying behavioral challenges experienced by children with ASD [53, 54]. The pattern characterized by limitations in social engagement and exploration may indicate difficulties in initiating and maintaining social connection, interpreting nonverbal signals, and exploring social environments. Impaired intrinsic motivations, such as reduced exploratory behavior and curiosity (Items 16, 15), as well as challenges in initiating social interactions (Item 13), are common in this pattern [55]. In addition, attention deficits (Item 18), a preference for solitary activities, and difficulties with self-care further indicate challenges in social participation and engagement.

Challenges in emotional awareness and cognitive responsiveness may reflect difficulties in understanding social contexts and interpreting others’ thoughts, emotions, and behaviors. The difficulty of recognizing facial expressions (Item 7) and comprehending verbal explanations (Item 9) suggest deficits in emotional communication and cognitive processing [56]. Furthermore, unaware of danger (Item 11), difficulty interpreting environmental cues (Item 10), and limited imagination (Item 12) indicate deficits in imaginative thinking [57]. These challenges are often linked to reduced emotional and cognitive flexibility, affecting abilities such as pretend play and imaginative engagement.

The pattern involving challenges in story comprehension and creativity suggests underlying problems in learning and sharing experiences. In an individual with ASD, the limitation in understanding TV demonstrations (Item 8) and inappropriate interactions with toys (Item 6) can reflect challenges in imaginative play and storytelling [58]. Limited participation in creative activities such as drawing and coloring (Item 5) further illustrates challenges in abstract thinking and understanding the motives of the character in stories [59].

Difficulties in social reciprocity and rewards highlight impairments in social exchanges and reduced pleasure derived from social interactions. Challenges in responding to praise (Item 2) and in recognizing one’s own name (Item 1) may indicate difficulties in perceiving and valuing social cues. These observations are supported by the social motivation theory of autism proposed by Chevallier et al. [60]. , which links social engagement difficulties to decreased sensitivity to social rewards. The fifth pattern, which is unique in terms of focus and attention, reflects the challenges of maintaining concentration, organizing tasks, and completing activities. Indicators such as reduced eye contact with people and animals (Item 3) and appearing “spacey” rather than attentive (Item 17) highlight challenges in focusing and committing to tasks [61, 62].

In the descriptions given above, the correlated variables in each pattern of sensory and cognitive impairments are likely linked to underlying shared atypical brain development [40, 63]. Limitations in social engagement, emotional awareness, and cognitive responsiveness may be linked to dysfunctions in the amygdala and the mirror neuron system [64, 65]. Decreased cognitive responsiveness has also been associated with abnormalities in frontal-striatal pathways and reduced activation in the anterior insula and prefrontal cortex [66–68]. Difficulties in story comprehension and creativity may be related to disrupted brain network integration and decreased white matter integrity [69, 70]. Atypical function within the ventral striatum and orbitofrontal cortex likely contributes to impaired social reciprocity and reward awareness [71]. Similarly, attentional deficits are frequently associated with altered activity in the anterior cingulate cortex (ACC) and dorsolateral prefrontal cortex (DLPFC) [72, 73].

While distinct sensory-cognitive impairment patterns are likely rooted in atypical neurodevelopmental origins, in Ethiopia, sociocultural and environmental factors could influence behavioral manifestations. Stigma-driven social isolation [74–77] and parental belief autism etiologies, such as God’s will, punishment for sin and evil eye (Buda), may contribute to observable behavioral patterns. Furthermore, belief in bad spirit possession and witchcraft can further influence these sensory and cognitive impairments. Traditional practices, such as Tsebel (Holy water), herbal remedies, and religious healing, remain common. These beliefs, along with widespread stigma and treatment options, often delay medical help-seeking and direct families toward spiritual or traditional healing over biomedical interventions [78].

Additionally, maternal stress caused by resource constraints, limited social support, and caregiving burdens may increase the severity of behavioral and functional impairments in children with ASD [79–81]. Furthermore, limited awareness at the individual and community levels can contribute to misinterpretations of ASD-related behaviors, potentially contributing to these patterns of sensory and cognitive awareness impairments.

## Clinical implications

This study establishes a culturally relevant factorial analysis of patterns of sensory and cognitive awareness impairments in the Ethiopian context. Identifying relevant patterns may help clinicians, therapists, educators, and caregivers apply targeted, evidence-based interventions specific to those patterns. Structured, guided conversation scripts, delivered through video modeling [82, 83] and reinforcement-based exploration protocols (engineered curiosity spaces) [84], can be used to address limitations in social engagement and exploration. Additionally, task-based self-care activities, such as hygiene practices and eating, might also be helpful, as they encourage social interaction and sensory regulation [85, 86]. Cultural activities and community market visits further increase functional improvements in both social engagement and exploratory behaviors [87].

In addressing challenges related to emotional awareness and cognitive responsiveness, clinicians and therapists can employ multimodal emotion activities [88], such as mirror-mediated exercises with facial expressions and clay modeling of faces. Practicing “pause-and-listen” activities paired with local hazard symbols can help increase danger and environmental awareness. Additionally, culturally contextualized structured imagination exercises, such as “what-if” scenario role-playing, may strengthen imaginative thinking and cognitive flexibility [89, 90].

Challenges in story comprehension and creativity manifested as impaired interpretation of TV narratives, limited symbolic play with toys, and reduced engagement in art activities may be improved through a structured video segmentation approach [91, 92]. Additionally, demonstrating tasks in real life along with visual picture cards that correspond to the video activity can further enhance story comprehension. Individualized reward pairing, which combines social praise and appreciation, can enhance challenges in social reciprocity and focus [93]. Furthermore, creating low-sensory work areas and minimizing environmental noise can enhance focus and attention.

## Limitations

This study provides scientific data on the patterns of sensory and cognitive awareness impairments among children diagnosed with ASD at autism centers in Addis Ababa, Ethiopia. However, the following limitations should be considered. First, while exploratory factor analysis was appropriate for this specific context, the findings may not fully represent all regions of Ethiopia, particularly rural areas. Patterns 4 and 5 showed high item loadings but had fewer items, raising concerns about their reliability. These findings suggest that these patterns may be less stable than larger factors are, and further studies with larger sample sizes are needed to ensure reliability and validity. Another limitation is that the data were collected through caregiver reports via the ATEC. Responses may have been influenced by factors such as caregiver perceptions, knowledge of ASD, stress levels, cultural interpretations of behavior, and recall bias. A study focused solely on children diagnosed with ASD in autism centers; excluding those from the broader community was a related limitation. Finally, the study was case-based, making it difficult to infer the underlying mechanisms contributing to the observed outcomes. Future research should adopt longitudinal or mixed-methods approaches to explore these mechanisms more effectively.

## Conclusion

Sensory and cognitive awareness impairments can be represented by five pattern structures. Clinicians and therapists may need to consider these patterns for more personalized and effective support of children with autism spectrum disorders.

## Data Availability

No datasets were generated or analysed during the current study.

## Abbreviations

ASD: Autism spectrum disorder
ATEC: Autism Treatment Evaluation Checklist
PCA: principal component analysis
KMO: Kaiser‒Meyer‒Olkin
